# Prevalence and Predictors of Scoliosis and Back Pain in 591 Adolescents: A Randomized, Stratified, Cross-Sectional Study in Riyadh, Saudi Arabia

**DOI:** 10.7759/cureus.26478

**Published:** 2022-07-01

**Authors:** Suhail S AlAssiri, Sami I Aleissa, Ali A Alhandi, Faisal M Konbaz, Fahad Alhelal, Majed Abaalkhail, Monerah M Al-Annaim, Abdulrahman Alhabeeb, Khaled M Alshehri

**Affiliations:** 1 Orthopaedic Surgery, Ministry of the National Guard-Health affairs, King Abdulaziz Medical City, King Abdullah International Medical Research Centre, and King Saud bin Abdulaziz University for Health Sciences, Riyadh, SAU; 2 Orthopaedic Surgery, Ministry of the National Guard-Health Affairs, King Abdulaziz Medical City, King Abdullah International Medical Research Centre, and King Saud bin Abdulaziz University for Health Sciences, Riyadh, SAU; 3 Orthopaedic Surgery, King Fahad Central Hospital, Ministry of Health, Jazan, SAU; 4 Medicine and Surgery, King Abdullah International Medical Research Centre and King Saud Bin Abdulaziz University for Health Sciences College of Medicine, Riyadh, SAU; 5 Orthopaedic Surgery, King Salman Armed Forces Hospital, Tabuk, SAU

**Keywords:** students, screening, prevalence, back pain, idiopathic scoliosis, adolescent

## Abstract

Purpose

Owing to the difficulty of establishing a screening program for scoliosis and back pain, along with their disabling consequences and the lack of local prevalence rates, we sought to study the prevalence of scoliosis and back pain in adolescents in Saudi Arabia and the burden reported by the affected age group on the health system.

Materials and methods

A school-based, cross-sectional pilot study covering all school districts in Riyadh, Saudi Arabia, was conducted. Students between 12 and 18 years of age were included. Students with any spinal or neurological disorders were excluded. Physical examinations to screen for scoliosis and student-filled questionnaires to assess back pain and health-related quality of life were performed.

Results

Of the 700 students, 591 met the inclusion criteria. High suspicion of adolescent idiopathic scoliosis (AIS) was considered in 174 students (29.44%). In addition, 45.42% of the students had a history of back pain. The Oswestry Disability Index showed that 87 students had disabilities. The average Scoliosis Research Society-22 score was 3 out of 5. A significant difference was found in the self-image and mental health domains for AIS (*p* = 0.04, *p* = 0.02, respectively). Age showed a significant increase in the odds ratio of a positive physical exam for every increase of one year in age (*p *< 0.01).

Conclusion

Identifying the prevalence rates and early associated factors during adolescence would help lower the burden on the health system and benefit public health in general. A nationwide study is required to identify the relationship between scoliosis and back pain.

## Introduction

Idiopathic scoliosis represents a common challenge that physicians face since it is a diagnosis of exclusion [1]. Infantile, juvenile, or adolescent idiopathic scoliosis (AIS) is classified based on the age at which it is first recognized [1-2]. AIS affects children over the age of 10, up to skeletal maturity, and with a prevalence of 0.47% to 5.2%, it is the most common spinal deformity that develops in otherwise healthy children [2-7]. Moreover, AIS has a female predominance with a 1.5:1 to 3:1 ratio [2]. As the name implies, the etiology of AIS is unknown, even though many theories have been proposed, including muscle imbalance and hormonal causes. Nevertheless, genetic factors may also play a role. To emphasize, 25% to 30% of AIS cases have a positive family history of scoliosis [2,8]. Unfortunately, some curves tend to progress, and untreated cases of AIS can increase morbidity and mortality. In addition, it can lead to many deleterious consequences, including lumbar radiculopathy and even cardiac and pulmonary restriction [2,8-10].

Many long-term follow-up studies have concluded that the appropriate management of AIS in childhood is associated with minimal pain and dysfunction later in life [10]. Furthermore, AIS reduces a patient's quality of life in a variety of ways, with the most common being back pain.

Many studies have shown that back pain is a widespread complaint associated with AIS, ranging from a 20% to 85% prevalence [2-6,11-12]. Regarding the intensity of the pain, it has been observed that higher levels of deformity are associated with more pain [6]. Additionally, back pain associated with AIS has been found to have a longer duration compared to that of non-scoliotic patients [12]. The lumbar spine is the most involved, followed by the thoracic spine [4,6,12-13]. Irrespective of whether it is acute or chronic, back pain has also been found to be linked with lower sleep quality and quantity [3]. The cause of back pain in adolescent population patients can originate from the facet joints, intervertebral discs, or sacroiliac joints [6]. Additionally, body image, which is a crucial matter at adolescent age, is also affected by AIS since it usually results in an obvious deformity. Unfortunately, it can impair social relations and affect the psychological well-being of children [14]. According to recent research, patients with AIS are more likely to experience depression and anxiety [14-15]. Back pain not only interferes with lifestyle and physical activity but also increases medication use, professional health care visits, and school absenteeism [6]. Establishing screening programs is a crucial step toward the early detection and possible conservative management of AIS. To accomplish this, comprehensive regional studies are required. AIS-related screening initiatives in Saudi Arabia are currently understudied. A few studies have assessed the population of highest risk and the epidemiological patterns of scoliosis, but unfortunately, there are no strong conclusions or recommendations related to screening programs [16-18].

Identifying local prevalence rates and instituting screening programs could significantly influence public health while also reducing the load on the health system. Because of the scarcity of local data on scoliosis screening programs and their efficacy, the argument over the need for and utility of comprehensive screening programs continues and no clear recommendation has been made. As a result, the purpose of this paper is to investigate the prevalence of scoliosis and back pain among adolescents in Saudi Arabia and the burden indicated by the afflicted age group.

## Materials and methods

Study design and settings

This school-based, cross-sectional pilot study was conducted in Riyadh, Saudi Arabia, covering all school districts in the city from January to April 2019. The target population was male and female students between the ages of 12 and 18 in Riyadh’s school system. Publicly available data from the Ministry of Education were acquired, and a population of 228,000 students was identified. Students with preexisting spine pathology, prior spine surgery, fractures in the spine, neurological disorders, or neuromuscular disorders were excluded from the study.

Population and study sample

A sample size of 384 students was the minimum required for prevalence calculations of unknown AIS prevalence in the region. The following formula was used for the sample size calculations where P is the estimated prevalence of 50%, Z is the equivalent score corresponding to the 95% confidence interval, and d is the margin of error of 5%.

Accounting for erroneously completed and missing questionnaires and students who refused to participate in the study, a target of 700 students was initially set. Stratified cluster random sampling was utilized for this study. A good representation of males and females in private and public schools was achieved through stratification. Classes within selected schools were randomly selected, and the whole class was invited to participate as a cluster.

Study instruments

AIS is diagnosed through radiological diagnosis, but it was not feasible to ask every student enrolled in the study to undergo a full-spine X-ray. A physical exam for shoulder and hip asymmetry is an alternative. Adams’s forward bending test was also used to detect the presence of a hump, along with scoliometer measurements to screen students with a high suspicion of scoliosis.

A 5° or more measurement was determined to be the cutoff for a positive test for high suspicion of AIS, which is the accepted standard practice [19]. AIS is a multifactorial disease that causes significant pain, with multiple possible effects on health-related quality of life (HRQL). Two questionnaires were administered to the enrolled students to assess both aspects: the Oswestry Disability Index (ODI) for back pain and the Scoliosis Research Society-22 (SRS22) questionnaire for HRQL. Both questionnaires were translated into Arabic and validated [20-21].

The ODI comprises 10 sections (domains), each having a total possible score of five (domains and scoring interpretations are detailed in Appendix A). The SRS22 consists of five domains, with a number of questions in each domain: five in function or activity, five in pain, five in self-image or appearance, five in mental health, and two in satisfaction with management. The satisfaction with management domain is a subsection that can be omitted and scored independently; this study omitted this last section because the students were not undergoing treatment (the scoring method is available in Appendix B). General participant data, such as age, gender, age of menarche when applicable, body mass index (BMI), history of back pain, and family history of scoliosis, were also acquired. The data collection was conducted by senior medical students supervised by the study co-investigators.

Statistical analysis

The data were entered into Excel (Microsoft Corporation, Redmond, WA) and imported to STATA software (StataCorp, 2015; Stata Statistical Software: Release 14. College Station, TX: StataCorp LP) for further analysis. Means and standard deviations were used for continuous variables, and percentages were reported for categorical variables. A multivariate logistic regression model was used to test associations between independent variables (age, gender, BMI, and history of back pain) and the number of students positive for AIS in a physical exam, which is the dependent variable. The logistic regression results were reported as odds ratio, with a p-value less than or equal to 0.05 as the cutoff for significance.

For the HRQL assessment, a comparison between students suspected to be scoliotic, as per this study’s parameters, and normal students was made by comparing the mean scores of both questionnaires. The Shapiro-Wilk test was done to test the normalization of data. The student’s t-test was used for normally distributed datasets, and the Mann-Whitney test was used for nonparametric data sets. A p-value of less than or equal to 0.05 indicated significant differences.

Ethical considerations

Institutional review board approval required student and parental consent, obtained after distributing letters of information to the students, parents, and school principals (RC 18/062/R).

## Results

Of the 700 students, 672 agreed to participate in the study, of which 591 met the inclusion criteria and completed the questionnaires. The male-to-female ratio was 10:8 (327 females and 264 males). The mean age was 15 ± 2 years, and the mean age of menarche was 13 years (9-16 years). The mean BMI was 22.72 ± 5.5 (10.9-46.1]. A family history of scoliosis was found in 45 students, with 41 in first-degree relatives. A positive history of back pain was noted in 268 students (45.42%).

Shoulder asymmetry in 149 students was noted during the physical examination, with 50 having pelvic asymmetry. Adam’s forward bending test showed a positive hump in 188 students, with 46 students having associated shifts to either side. Only 174 students (29.44%) were considered positive for high suspicion of AIS with scoliometer measurements, as defined by this study. Further assessment of HRQL utilized the ODI and SRS22 instruments. The ODI instrument found 71 students to have moderate disabilities and 15 students to have severe disabilities, and one student was considered crippled by the instrument. No statistically significant differences were noted between the two groups. The average total score of the SRS22 was 3 out of 5 for the sample. A comparison between the means of the two groups was made for the average total and the subtotals of each subcategory in the questionnaire. No differences were noted in the function and pain subcategories (p = 0.6763 and p = 0.4649, respectively). However, the self-image and mental health subcategories showed significant differences between students considered positive for AIS and those who were not. (p = 0.04 and p = 0.02, respectively). No statistically significant difference in the average total score between the two groups was noted. Further analysis with a regression model found a positive association between AIS in the physical exam and the female gender, with an odds ratio of 4.08 and a p-value lower than 0.01.

Moreover, age showed a significant increase in the odds ratio of a positive physical exam for every gain by one year, with an odds ratio of 1.18 and a p-value lower than 0.01. The other tested variables were statistically insignificant (Table 1).

**Table 1:** Multivariant regression analysis model testing significant variables against a positive Adam’s forward bend test as a dependent variable *Std. Err. = standard error Model fit measures: Log likelihood = -325.66478, Pseudo R squared = 0.0899

Variable	Odds Ratio	Std. Err.*	Z score	p-value	(95% Conf. Interval)	
Age	1.184634	0.0641645	3.13	0.002	1.06532	1.317312
Gender	4.086895	0.857675	6.71	0.000	2.708698	6.166326
BMI	0.9975351	0.0039885	-0.62	0.537	0.9897484	1.005383
Back pain	1.104047	0.2146647	0.51	0.611	0.7541967	1.616182

## Discussion

Scoliosis is defined as a deviation from the normal spine, including a lateral curvature with a rotated vertebra within the curve [22]. Many studies have found back pain to be the most common presenting symptom in scoliotic patients. While many patients and their families assume that the pain is caused by deformities, there is no scientific evidence linking back pain and scoliosis [11-12].

Although the extent of back pain and scoliosis burden has not yet been well-defined, half of our population was considered disabled based on the ODI in our study [23]. Likewise, Wong et al. examined the impact of back pain and biopsychosocial variables on AIS patients. Compared to the general population, patients had significantly higher levels of clinical anxiety, depression, insomnia, daytime sleepiness, back pain, and physical strain. These psychological symptoms were also correlated with Cobb angles greater than 30 degrees, female gender, and older age [3].

Furthermore, we omitted the last section of the SRS22 because the students would not be managed. On the other hand, we tried to assess many aspects of scoliotic patients, including their characteristics, disability, function, pain, self-image, and mental health. Our study found a very high number of students with a positive history of back pain. In contrast to our study, Sato et al. found that scoliotic students experienced significantly severe pain with a longer duration in an epidemiological study of 43,630 students [12]. A possible explanation for this contradiction is the sample size. The study also found that the lifetime prevalence of back pain in scoliotic patients was 58.8%, a two-fold higher odds ratio than the non-scoliotic group [12]. However, Balagué and Pellisé looked into scoliosis and back pain in the literature and concluded that back pain is a common complaint in this age group, and there was no evident correlation between deformity and back pain [11]. In contrast to our study, Lonner et al. utilized the SRS22 questionnaire and found significant differences between the back pain scores of scoliotic and healthy controls. Consistently, in the self-image and mental health domains, it was found in the study of Lonner et al. and our study that AIS patients had significantly lower scores in both domains [24]. We also found a remarkable number of participants who reported a positive family history of scoliosis. This is following the fact that many studies have suggested the possibility of genetics being a cause of AIS [2,8,25].

In terms of screening programs, Fong et al. looked into the effectiveness and suitability of Hong-Kong screening programs for scoliosis with a large population study and a follow-up period of 10 years. The results showed the effectiveness of the screening program with the achievement of its goal, sensitivity, and specificity of around 70% [26]. Likewise, The American Academy of Orthopedic Surgery, SRS, Pediatric Orthopedic Society of North America, and Academy of Pediatrics advocated for larger studies to find evidence relating to the effectiveness of scoliosis screening programs. They reviewed the available data regarding the benefits and harms of screening programs for adolescents aged 10-18 years old. The rationale behind screening was to recognize and treat scoliosis at earlier stages and slow the curve progression before skeletal maturity, possibly improving long-term outcomes. However, limited evidence was available to conclude the effectiveness of screening, especially for long-term outcomes [27]. In addition, the United States Preventive Services Task Force (USPSTF) concluded in their recommendations that the current evidence is insufficient and that there are no benefits to the patient-centered module [23]. After implementing screening programs, the number of false-positive referrals would increase, which could lead to psychological harm and unnecessary radiation exposure and treatment [26-28]. On the other hand, there was adequate evidence of health benefits in a small percentage of the population, including decreased pain and disability. Finally, the balance between the benefits and risks of screening remains undetermined [27].

Based on a review of seven studies, the prevalence of AIS can vary from 0.47% to 5.2% [7]. However, in our study, 29.44% of the students had a high suspicion of AIS according to scoliometer measurements. This large difference can be attributed to our study's lack of radiological assessment. In Saudi Arabia, some studies have discussed the prevalence of AIS and the need for screening programs. One study screened 1147 schoolgirls and found the prevalence of AIS to be 2.5% [16]. In another study that included 1117 boys, it was found that the prevalence of AIS was only 0.78% [17]. The low percentage could be explained by the inclusion of only boys in their study.

## Conclusions

In our study, we provide a more comprehensive view of AIS, including the psychological impact and the back pain caused by it. Moreover, we included both genders and assessed their disability and quality of life. Identifying local prevalence rates and possible early related factors during adolescence has a significant impact on public health, not to mention the reduced cost on the health system. Our study was limited by its cross-sectional design, as we could not assess the progression of scoliosis. Moreover, given that AIS is a radiological diagnosis, the study was limited because it was not possible to diagnose the participants radiologically. Instead, we relied entirely on history and physical examination. However, participants who needed additional images for confirmatory diagnosis were advised to approach primary health care providers for a routine check-up. In conclusion, we believe that large-scale screening and investigation are needed to determine the true prevalence of back pain and scoliosis and the link between them.

## Figures and Tables

**Figure 1:**
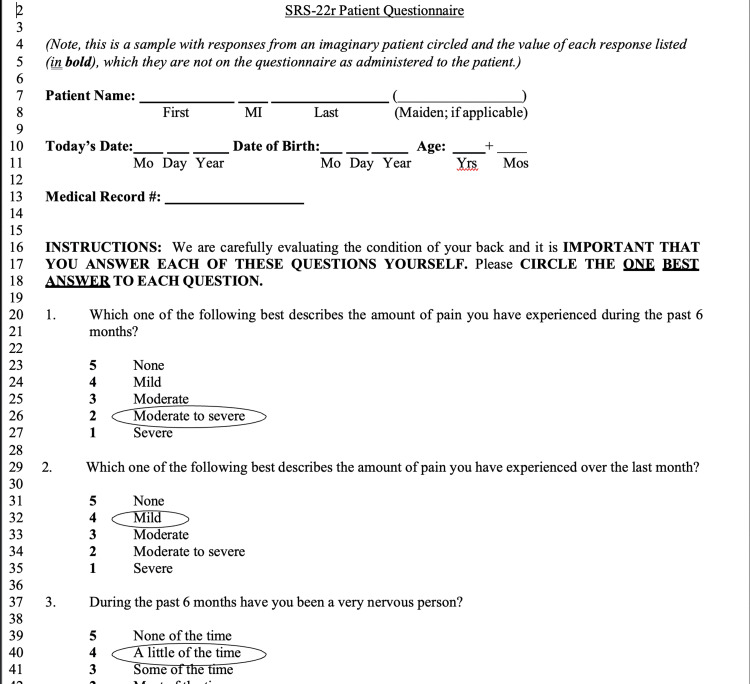
SRS-22 patient questionnaire SRS-22 = Scoliosis Research Society-22

## Appendices

Appendix A

Oswestry Low Back Pain Disability Questionnaire

Sources: Fairbank JCT & Pynsent, PB (2000) The Oswestry Disability Index. Spine, 25(22):2940-2953.

Davidson M & Keating J (2001) A comparison of five low back disability questionnaires: reliability and responsiveness. Physical Therapy 2002;82:8-24.

The Oswestry Disability Index (also known as the Oswestry Low Back Pain Disability Questionnaire) is an extremely important tool that researchers and disability evaluators use to measure a patient's permanent functional disability. The test is considered the ‘gold standard’ of low back functional outcome tools [1].

Scoring instructions: For each section, the total possible score is 5: if the first statement is marked the section score = 0; if the last statement is marked, it = 5. If all 10 sections are completed the score is calculated as follows:

Example: 16 (total scored)

50 (total possible score) x 100 = 32%

If one section is missed or not applicable, the score is calculated:

16 (total scored)

45 (total possible score) x 100 = 35.5%

Minimum detectable change (90% confidence): 10% points (change of less than this may be attributable to an error in the measurement)

**Table 2:** Interpretation of scores

0% to 20%: minimal disability:	The patient can cope with most living activities. Usually, no treatment is indicated apart from advice on lifting, sitting, and exercise.
21%-40%: moderate disability:	The patient experiences more pain and difficulty with sitting, lifting, and standing. Travel and social life are more difficult and they may be disabled from work. Personal care, sexual activity, and sleeping are not grossly affected and the patient can usually be managed by conservative means.
41%-60%: severe disability:	Pain remains the main problem in this group but activities of daily living are affected. These patients require a detailed investigation.
61%-80%: crippled:	Back pain impinges on all aspects of the patient's life. Positive intervention is required.
81%-100%:	These patients are either bed-bound or exaggerating their symptoms.

Oswestry Low Back Pain Disability Questionnaire

Instructions: This questionnaire has been designed to give us information as to how your back or leg pain is affecting your ability to manage in everyday life. Please answer by checking ONE box in each section for the statement which best applies to you. We realize you may consider that two or more statements in any one section apply but please just shade out the spot that indicates the statement that most clearly describes your problem.

**Table 3:** Questionnaire

Section 1 – Pain intensity
I have no pain at the moment
The pain is very mild at the moment
The pain is moderate at the moment
The pain is fairly severe at the moment
The pain is very severe at the moment
The pain is the worst imaginable at the moment
Section 2 – Personal care (washing, dressing, etc.)
I can look after myself normally without causing extra pain
I can look after myself normally but it causes extra pain
It is painful to look after myself and I am slow and careful
I need some help but manage most of my personal care
I need help every day in most aspects of self-care
I do not get dressed, I wash with difficulty and stay in bed

Section 3 – Lifting
I can lift heavy weights without extra pain
I can lift heavy weights but it gives extra pain
Pain prevents me from lifting heavy weights off the floor, but I can manage if they are conveniently placed eg. on a table
Pain prevents me from lifting heavy weights, but I can manage light to medium weights if they are conveniently positioned
I can lift very light weights
I cannot lift or carry anything at all
Section 4 – Walking*
Pain does not prevent me from walking any distance
Pain prevents me from walking more than 1 mile
Pain prevents me from walking more than 1/2 mile
Pain prevents me from walking more than 100 yards
I can only walk using a stick or crutches
I am in bed most of the time

Section 5 – Sitting
I can sit in any chair as long as I like
I can only sit in my favorite chair as long as I like
Pain prevents me from sitting for more than one hour
Pain prevents me from sitting for more than 30 minutes
Pain prevents me from sitting for more than 10 minutes
Pain prevents me from sitting at all
Section 6 – Standing
I can stand as long as I want without extra pain
I can stand as long as I want but it gives me extra pain
Pain prevents me from standing for more than 1 hour
Pain prevents me from standing for more than 30 minutes
Pain prevents me from standing for more than 10 minutes
Pain prevents me from standing at all
Section 7 – Sleeping
My sleep is never disturbed by pain
My sleep is occasionally disturbed by pain
Because of pain, I have less than 6 hours of sleep
Because of pain, I have less than 4 hours of sleep
Because of pain, I have less than 2 hours of sleep
Pain prevents me from sleeping at all

Section 8 – Sex life (if applicable)
My sex life is normal and causes no extra pain
My sex life is normal but causes some extra pain
My sex life is nearly normal but is very painful
My sex life is severely restricted by pain
My sex life is nearly absent because of pain
Pain prevents any sex life at all
Section 9 – Social life
My social life is normal and gives me no extra pain
My social life is normal but increases the degree of pain
Pain has no significant effect on my social life apart from limiting my more energetic interests eg, sport
Pain has restricted my social life and I do not go out as often
Pain has restricted my social life to my home
I have no social life because of pain
Section 10 – Travelling
I can travel anywhere without pain
I can travel anywhere but it gives me extra pain
Pain is bad but I manage journeys over two hours
Pain restricts me to journeys of less than one hour
Pain restricts me to short necessary journeys under 30 minutes
Pain prevents me from traveling except to receive treatment

References

1. Fairbank JC, Pynsent PB. The Oswestry Disability Index. Spine 2000 Nov 15;25(22):2940-52; discussion 52.

Appendix B
